# MODULATING CELLULAR SENESCENCE AND MTOR PATHWAY: THE THERAPEUTIC POTENTIAL OF FINGOLIMOD IN NPC DISEASE

**DOI:** 10.1093/geroni/igae098.3675

**Published:** 2024-12-31

**Authors:** Morteza Abyadeh, Alaattin Kaya

**Affiliations:** Virginia Commonwealth University, Richmond, Virginia, United States; Virginia Commonwealth University, Richmond, Virginia, United States

## Abstract

Niemann-Pick type C (NPC) disease is a genetically-driven neurodegenerative disorder with no effective treatment. Neurodegeneration, particularly in the cerebellum, is a key pathological feature. Fingolimod phosphate (FTY720), initially approved for multiple sclerosis, functions mainly as an immunomodulator but shows potential in treating neurodegenerative conditions. This study investigates FTY720’s therapeutic potential in mitigating cellular senescence and neurodegeneration in NPC using both in vitro and in vivo models. Our findings reveal that FTY720 pretreatment reduces cellular senescence in fibroblasts, delays muscle mass decline, and improves motor neuron function in NPC mouse models, as evidenced by performance in balance beam and vertical screen tests. Omics analyses indicate that FTY720 exerts anti-inflammatory and anti-aging effects by modulating mTOR signaling and complement coagulation pathways. Importantly, FTY720’s effects were sex-specific, with greater efficacy observed in male mice. Immunohistopathological studies further confirmed FTY720’s protective effects, showing more pronounced benefits in males. In conclusion, FTY720 demonstrates significant promise as a treatment for NPC, particularly in males, by delaying disease symptoms through its protective effects against cellular senescence and inflammation.

